# Hamlet-Pattern-Based Automated COVID-19 and Influenza Detection Model Using Protein Sequences

**DOI:** 10.3390/diagnostics12123181

**Published:** 2022-12-15

**Authors:** Mehmet Erten, Madhav R. Acharya, Aditya P. Kamath, Niranjana Sampathila, G. Muralidhar Bairy, Emrah Aydemir, Prabal Datta Barua, Mehmet Baygin, Ilknur Tuncer, Sengul Dogan, Turker Tuncer

**Affiliations:** 1 Laboratory of Medical Biochemistry, Malatya Training and Research Hospital, 44000 Malatya, Turkey; 2 Department of Biomedical Engineering, Manipal Academy of Higher Education, Manipal 04478, India; 3 Center for Biomedical Engineering, Brown University, Providence, RI 02912, USA; 4 Department of Management Information, College of Management, Sakarya University, 54050 Sakarya, Turkey; 5 School of Management & Enterprise, University of Southern Queensland, Toowoomba, QLD 4350, Australia; 6 Faculty of Engineering and Information Technology, University of Technology Sydney, Sydney, NSW 2007, Australia; 7 Department of Computer Engineering, Faculty of Engineering, Ardahan University, 75000 Ardahan, Turkey; 8 Elazig Governorship, Interior Ministry, 23119 Elazig, Turkey; 9 Department of Digital Forensics Engineering, Technology Faculty, Firat University, 23119 Elazig, Turkey

**Keywords:** Hamlet Pattern, protein sequence classification, SARS-CoV-2, bioinformatics

## Abstract

SARS-CoV-2 and Influenza-A can present similar symptoms. Computer-aided diagnosis can help facilitate screening for the two conditions, and may be especially relevant and useful in the current COVID-19 pandemic because seasonal Influenza-A infection can still occur. We have developed a novel text-based classification model for discriminating between the two conditions using protein sequences of varying lengths. We downloaded viral protein sequences of SARS-CoV-2 and Influenza-A with varying lengths (all 100 or greater) from the NCBI database and randomly selected 16,901 SARS-CoV-2 and 19,523 Influenza-A sequences to form a two-class study dataset. We used a new feature extraction function based on a unique pattern, HamletPat, generated from the text of Shakespeare’s *Hamlet*, and a signum function to extract local binary pattern-like bits from overlapping fixed-length (27) blocks of the protein sequences. The bits were converted to decimal map signals from which histograms were extracted and concatenated to form a final feature vector of length 1280. The iterative Chi-square function selected the 340 most discriminative features to feed to an SVM with a Gaussian kernel for classification. The model attained 99.92% and 99.87% classification accuracy rates using hold-out (75:25 split ratio) and five-fold cross-validations, respectively. The excellent performance of the lightweight, handcrafted HamletPat-based classification model suggests that it can be a valuable tool for screening protein sequences to discriminate between SARS-CoV-2 and Influenza-A infections.

## 1. Introduction

Understanding how viruses interact with host cells for their life cycle is essential for understanding viral pathogenesis. In addition, the viral genetic codes enable these interactions. The unique protein sequences that distinguish between viruses are a crucial cornerstone. For this purpose, medical laboratories make great efforts. On the other hand, in silico approaches cover a wider place in today’s academic research than ever before. This study proposes a method based on viral protein sequences that can classify SARS-CoV-2, which has scared the world in recent years, and the Influenza-A virus, which causes difficulties in differential diagnosis [1].

SARS-CoV-2 is a new type of coronavirus that emerged in December 2019, causing severe acute respiratory distress syndrome [1]. It spread worldwide, resulting in a pandemic named COVID-19. Coronaviridae family members are enveloped by single-stranded positive-sense Ribonucleic acid (RNA) viruses [2]. SARS-CoV-2 [3] shares a 50% similarity with the MERS-CoV genome. Additionally, it has 88% similarity to two SARS-like coronaviruses derived from bat species [4]. The SARS-CoV-2 genome contains 14 open reading frames (ORFs) that encode about 27 proteins [5], including four structural (surface (spike) (S), envelope (E), matrix (membrane) (M)) proteins, and a nucleocapsid (N) protein [5]. The S protein binds to host cells and exhibits tropism towards the angiotensin-converting enzyme receptors (ACE-2s) [6]. ACE-2 is highly expressed in alveolar type 2 epithelial cells, which explains the vulnerability to pneumonia [7]. The S protein consists of two subunits: S1 binds to the receptor on the host cell membrane, while S2 fuses the viral and host membranes [8]. Therefore, these proteins are potential therapeutic drug targets [8,9].

The influenza virus is an RNA virus belonging to the Orthomyxoviridae family that can infect a variety of human and animal hosts. Influenza B and C are more common in humans, while Influenza D infects animals such as goats and pigs [10]. The genomes of Influenza A and B viruses contain eight RNA segments that encode 18 different proteins. Influenza C and D lack the fifth segment responsible for neuraminidase. The fourth gene encodes hemagglutinin, and other genes encode viral polymerases, nucleoproteins, major matrix protein, ion channel matrix protein, and other nonstructural proteins [11]. Influenza-A viruses are named according to the structural differences of neuraminidase (N1-11) and hemagglutinin glycoproteins (H1–18), which are on the surface of the envelope [3]. These envelope proteins play an important step in viral pathogenesis by binding to the host cells [12]. This stage is also an important drug target [13].

The viral pathophysiology that we previously mentioned, and the etiopathogenesis of influenza explained in the prior section, should illustrate why the viral genomes are the crucial component of these diseases and their therapies. SARS-CoV-2 and Influenza-A can present with similar symptoms, and Influenza-A infection may be more problematic for patients during the current COVID-19 pandemic [14]. In addition, computer-aided diagnosis could help screen for the two pathological conditions requiring different treatment and isolation protocols [15].

Our main aim is to propose a new machine learning model to classify COVID-19 and Influenza-A diseases with high classification performance. Moreover, we propose a lightweight protein classification model. This model uses a new feature extraction technique named Hamlet Pattern. We in fact propose a new feature extraction methodology, and Hamlet Pattern is the first feature generator of the presented feature extraction methodology. This methodology is named text-based feature extractor creation. To take attention, we have used the popular text, *Hamlet*. In the Materials and Methods we present the main steps in the creation of our text-based feature extractor. Our main hypothesis is that literary texts have harmonies, which have been created using hidden patterns. In this paper we propose a new feature extraction methodology to obtain these hidden patterns. We used a piece of text from *Hamlet* to generate a new pattern, and the features were extracted using this pattern. We tested the feature generation ability of the presented feature extraction function using a protein sequence dataset to classify COVID-19 and Influenza-A.

Machine learning and artificial intelligence models are commonly used in the biomedical and bioinformatics sciences to solve classification problems [16,17,18]. Therefore, we were motivated to develop a computationally lightweight machine learning model for automated SARS-CoV-2 versus Influenza-A diagnosis. Feature engineering is an important aspect of machine learning [19,20]. One popular handcrafted feature generator is the local binary pattern (LBP) [21], which extracts local textual features based on the neighborhood relations of overlapping blocks. LBP possesses the advantages of simple application, low time complexity, ability to generate distinctive features, and fixed-size feature vector creation. We were motivated to develop an LBP-like feature extractor based on a novel pattern inspired by art. To this end, we selected text from a famous literary work, *Hamlet*, to create a novel pattern, combined with the feature selection function and a standard shallow classifier to form a handcrafted learning model.

The contributions of the proposed model are as follows:

- A novel feature extraction method based on a novel pattern that was inspired by a literary work. The presented feature extraction method is the first text-based feature extraction function creation methodology.
- Using protein sequences, a classification model incorporating the novel pattern was applied for the binary classification of SARS-CoV-2 versus Influenza-A diagnosis. The model attained excellent classification performance, supporting its potential use as an adjunctive screening tool for suspected viral respiratory infections in the current pandemic.

The paper is organized as follows: The dataset is shown in Section 2. Section 2 describes our proposed protein sequence classification model. Section 3 presents the results. Section 4 presents a discussion of the results. Section 5 outlines the conclusions.

## 2. Materials and Methods

### 2.1. Materials

In FASTA format, we downloaded viral protein sequences of SARS-CoV-2 and Influenza-A with lengths 100 or greater from the NCBI database [22]. Among these, we randomly selected 16,901 SARS-CoV-2 and 19,523 Influenza-A sequences to form a two-class study dataset with 36,424 observations.

### 2.2. Our Proposed Protein Sequence Classification Model

**Figure 1:**
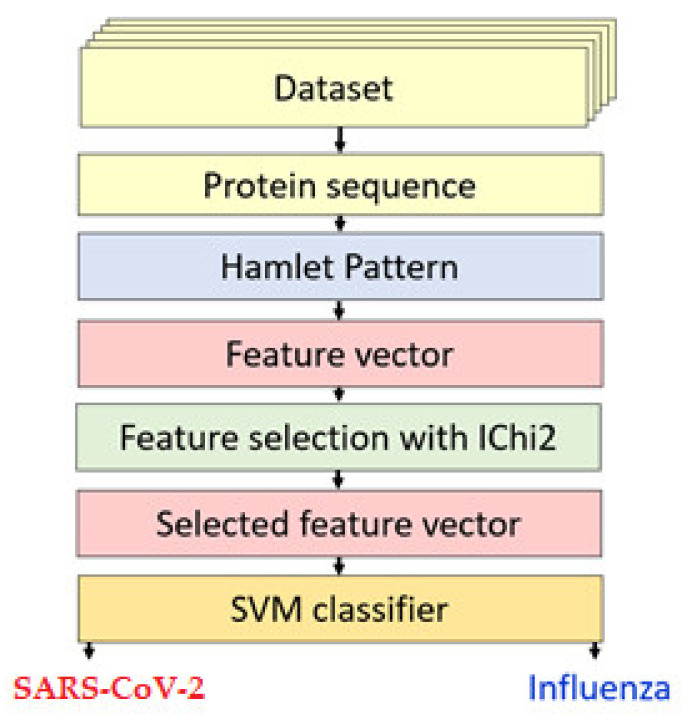
Schema of the proposed HamletPat-based model for binary classification of viral protein sequences.

Shakespeare’s *Hamlet* inspired our novel handcrafted feature engineering method. In Act 1 Scene 1, an apparition is before Horatio, who initially doubts but later acknowledges it to be the ghost of the recently deceased King Hamlet [23]. Horatio, the speaker of truth, i.e., the oracle, and confidant of the protagonist Prince Hamlet, later informed him of the encounter, thus setting the unfolding of subsequent tragic events in motion. By using letters from this text, HamletPat is applied to extract features from protein sequences. The protein sequences were coded with letters because the amino acids are named with text. We converted these texts to numerical values. HamletPat extracts a feature vector from these numerical values. An iterative Chi-square (IChi2) application [24] was then deployed to choose the most discriminative features to feed a support vector machine (SVM) [25,26] for classification using hold-out (75:25 split ratio) as well as 5-fold cross-validations (CVs) (see Figure 1).

The basic steps of the model are listed below (details are provided in the following sections):

*Step 0:* Load/read each protein sequence from the dataset and convert the amino acid sequence conventionally denoted by letters of the alphabet to a string of numbers.

*Step 1:* Extract features from each protein sequence using HamletPat.

*Step 2:* Select highly discriminative features from the generated feature vector by deploying the IChi2 feature selection function.

*Step 3:* Classify selected features by deploying the SVM classifier with two validation techniques, hold-out (split ratio 75:25) and 5-fold CVs.

#### 2.2.1. Feature Extraction Using HamletPat

**Figure 2:**
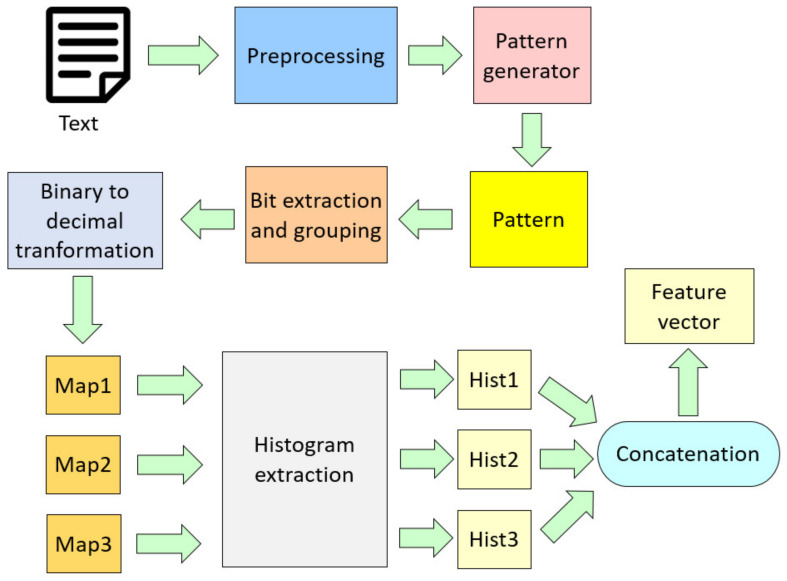
Block diagram of the proposed text-based feature extraction function generation model. We used *Hamlet* as a text in this paper. In the figure, Map defines feature map signals, and Hist is histogram.

A novel text-generated pattern, HamletPat, was used to extract LBP-like features from overlapping fixed-length blocks (27) of protein sequences of different lengths. A block diagram of the proposed feature extraction function is shown in Figure 2.

First, text from the first page of *Hamlet* Act 1 Scene 1 was pre-processed by deconstructing it into letters of the English alphabet, with special characters deleted and all uppercase letters converted to lowercase. Next, the letters were enumerated from 1 to 26 using ASCII conversion and input to a pattern generator algorithm. The output, HamletPat, was then used to extract bits, similar to LBP feature extraction, from overlapping fixed-length blocks of protein sequences of varying lengths to construct the final feature vector. The feature engineering steps are detailed below:

*1:* Choose the text. In this model, we chose the text from *Hamlet* Act 1 Scene 1.

*2:* Remove all special characters in the text.

*3:* Transform all uppercase letters to lowercase.

**Figure 3:**
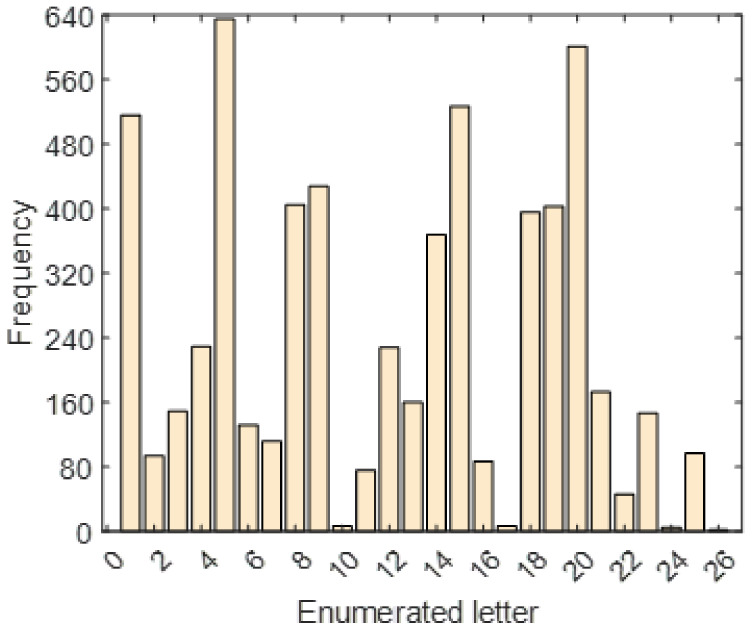
Frequency histogram of the enumerated letters used in the selected *Hamlet* text.

*4:* Enumerate letters by using their ASCII code.

(1) $$val=ascii\left(lc\right)-96$$

where val defines value and $ascii\left(.\right)$ is the ASCII value conversion function of lc, the lowercase character. The frequency histogram of the enumerated letters is shown in Figure 3.

*5:* Generate a pattern by deploying Algorithm 1.

**Algorithm 1.** Pattern generator using enumerated letters.**Input:** The calculated values of the letters **Output:** Pattern01: **for**
*i* = 1 to 26 **do** // Assign counter02:            $counter\left(i\right)=0$;03: **end for i**04: i = 1; j = 1; // Define variables05: $sum=\sum_{i=1}^{26}counter\left(i\right);$
06: **while** sum < 26 **do**07:            $v=val\left(i\right);$
08:            **if**
$counter\left(v\right)=0$
**then**09:                      $counter\left(v\right)=1$;10:                      $pattern\left(j\right)=v$;11:                      j++;
12:            **end if**13:            $sum=\sum_{i=1}^{26}counter\left(i\right);$
14:            i++;
15: **end while**

Computed values or indexes in the pattern array, which are arranged in ascending order of the identifying number of the enumerated letters (Table 1), constituted the HamletPat.

*6:* Divide the signal/sequence into overlapping blocks with a length of 27 each.

(2) $$b=s\left(j+i-1\right),\;j\in \left\{1,2,\ldots ,27\right\},\;i\in \left\{1,2,\ldots ,l-26\right\}$$

where s represents the utilized one-dimensional signal with a length of l and b represents the overlapping block with a size of 27. To create overlapping blocks, frameshift is defined as one (stride = 1). Therefore, l−26 overlapping blocks with a length of 27 were created from a one-dimensional signal/array with a length of l.

*7:* Choose the center value (14th value) as the center.

(3) $$c=b\left(14\right)$$

where c is the center value.

*8:* Enumerate all other values, skipping the 14th value.

(4) $$d\left(h\right)=b\left(h\right),\;h\in \left\{1,2,\ldots ,13\right\}$$

(5) $$d\left(k-1\right)=b\left(k\right),\;k\in \left\{15,16,\ldots ,27\right\}$$

where d represents renumbered values with a length of 26.

*9:* Create bits using the HamletPat, renumbered values, center value, and signum function.

(6) $$bf\left(i\right)=sign(center,d\left(pattern\left(i\right)\right),\;i\in \left\{1,2,\ldots ,26\right\}$$

(7) $$sign\left(q,w\right)=\left\{\begin{array}{c} 0,\;q-w\geq 0\\ 1,q-w<0 \end{array}\right.$$

where bf represents bits; $sign\left(.,.\right),$ signum function; and $\left(q,w\right)$, parameters of the signum function.

*10:* Divide the generated bits into three groups with bit lengths 9, 8, and 9, respectively.

(8) $$first\left(r\right)=bf\left(r\right),\;r\in \left\{1,2,\ldots ,9\right\}$$

(9) $$second\left(t\right)=bf\left(r+t\right),\;t\in \left\{1,2,\ldots ,8\right\}$$

(10) $$third\left(r\right)=bf\left(r+17\right)$$

*11:* Calculate three map signals from the three-bit groups using binary-to-decimal conversion.

(11) $$map_{1}\left(i\right)=\overset{9}{\underset{j=1}{\sum }}first\left(j\right)\times 2^{9-j}$$

(12) $$map_{2}\left(i\right)=\overset{8}{\underset{j=1}{\sum }}second\left(j\right)\times 2^{8-j}$$

(13) $$map_{3}\left(i\right)=\overset{9}{\underset{j=1}{\sum }}third\left(j\right)\times 2^{9-j}$$

*12:* Extract histograms ($hist_{1}$, $hist_{2}$, $hist_{3}$) from the corresponding $map_{1},map_{2}$, and $map_{3}$, which have lengths equal to 512 (=2^9^), 256 (=2^8^), and 512 (=2^9^), respectively.

*13:* Merge the generated histograms to obtain the feature vector, which has a length of 1280.

(14) $$ftvec\left(p\right)=hist_{1}\left(p\right),\;p\in \left\{1,2,\ldots ,512\right\}$$

(15) $$ftvec\left(z+512\right)=hist_{2}\left(z\right),\;z\in \left\{1,2,\ldots ,256\right\}$$

(16) $$ftvec\left(p+768\right)=hist_{3}\left(p\right)$$

#### 2.2.2. Iterative Chi-Square Feature Selection

For feature selection, we deployed IChi2, which uses the parametric Chi-square function, one of the fastest in the literature [27], to compute qualified indexes of the features. IChi2 is efficient at iteratively selecting highly discriminative features using fewer features, effectively reducing the execution times of the classifiers. An iteration range is typically set, and then variable feature vectors are selected iteratively using a loss value calculator. Here we set the iteration range at (100–500), and an SVM with a Gaussian kernel (see Section 3.3 below) was deployed as the loss calculator using a 5-fold CV. In our experiments on the study dataset, the SVM calculated the loss values of 401 (=500 – 100 + 1) feature vectors, and IChi2 selected the optimal feature vector of length 340.

#### 2.2.3. Classification

An SVM was deployed as a loss calculator (see Section 3.2 above) and classifier. The hyperparameters were set as: kernel function, Gaussian; kernel scale, 4.6; box constraint, one [25,26]. Both five-fold and hold-out CVs (split ratio 75:25, i.e., the dataset was randomly split into 75% and 25% for training and testing, respectively) were used for the classification task, whereas only the former was used for loss calculation during the IChi2 feature selection.

## 3. Results

### 3.1. Experimental Setup

The study dataset comprised two viral protein sequence classes, each typically notated as a string of letters corresponding to the individual amino acid’s building blocks. An ASCII code table was used to transform these letters into integer values. The latter were then input to the proposed handcrafted HamletPat-based model. The model was computationally lightweight and was implemented in a MATLAB (2021b) environment on a personal computer with an Intel i9-9900 Processor (cache 16 M, clock speed 5 GHz) and 48 GB memory, using Microsoft Windows 10.1 Professional operating system. The presented Hamlet-Pattern-based model has linear time complexity. Therefore, more simply configured computers can be used for implementation. There is no need to use expensive hardware, for instance, a graphical processing unit (GPU) and a tensor processing unit (TPU). Therefore, this model can be implemented on any computer. Moreover, the proposed Hamlet Pattern model can be embedded in a card.

### 3.2. Evaluation Metrics

Standard metrics were used to evaluate the performance of the model for binary classification: accuracy (ac), sensitivity (sn), specificity (sp), precision (pr), F-measure (f1), and geometric mean (geomean). The metrics were calculated from the numbers of true positive (tp), true negative (tn), false positive (fp), and false negative (fn) results using Equations (17) to (22) [28,29].

(17) $$ac=\frac{tp+tn}{tp+tn+fp+fn}$$

(18) $$sn=recall=\frac{tp}{tp+fn}$$

(19) $$sp=\frac{tn}{fp+tn}$$

(20) $$pr=\frac{tp}{tp+fp}$$

(21) $$f1=2\frac{pr\times sn}{pr+sn}$$

(22) $$geomean=\sqrt{sp\times sn}$$

### 3.3. Performance of the Proposed Model

The model’s performance metrics stratified by the validation schemes, i.e., hold-out versus five-fold CVs and virus type, are summarized in Table 2.

The model attained 99.92% and 99.87% classification accuracy using hold-out and five-fold CVs, respectively. Of note, 100% sensitivity for SARS-CoV-2 detection was achieved by deploying a hold-out CV with a 75:25 split ratio.

**Figure 4:**
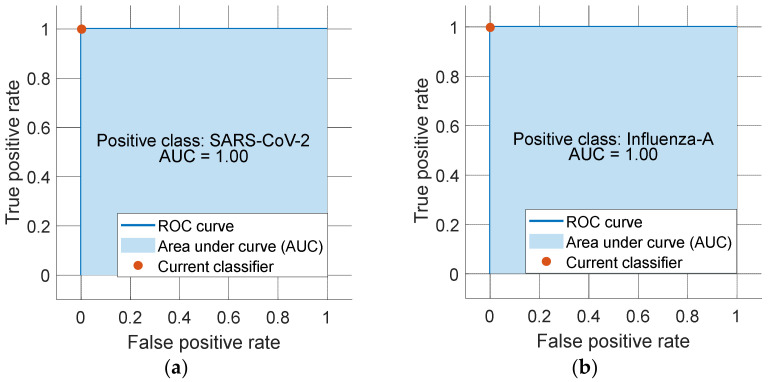
ROC curves of each class (**a**) SARS-CoV-2 and (**b**) Influenza-A.

ROC curves were added to evaluate this model. The ROC curves of both classes are demonstrated in Figure 4.

As can be seen from Figure 4, the proposed model attained a 100% area under curve (AUC) value.

### 3.4. Time Complexity Analysis

We calculated the time complexity of the proposed model and describe the results in this section. The presented Hamlet Pattern is a handcrafted model. Therefore, the time burden of this feature extractor is equal to $O\left(n\right)$. Herein, n is the length of the signal. To choose features, the IChi2 feature selector was used. IChi2 uses Chi2, a loop, and a loss value calculator. Therefore, it is calculated as $O\left(s+ic\right).$ The used s,i, and c variables are the time burden coefficients of the feature selector, the number of loops, and the time burden coefficients of the classifier, respectively. In the classification phase, a shallow classifier is used, and its computational complexity is $O\left(c\right)$. In total, the time complexity of the presented Hamlet-Pattern-based classification model is $O\left(n+s+ic+c\right)≅O\left(n+s+ic\right).$ This result demonstrates that the presented model has linear time complexity.

## 4. Discussion

SARS-CoV-2 and Influenza-A are very different pathogens that share important overlapping clinical features. In the current COVID-19 pandemic, SARS-CoV-2 has caused nearly six million deaths worldwide. The H1N1 Influenza-A virus was the cause of the Spanish flu that infected one-third of the world’s population and killed millions from 1918 to 1920 [30]. Subsequent influenza-related pandemics had less-severe consequences [31]. According to the World Health Organization, about half a million people, mostly the elderly, die from seasonal influenza cases each year [3]. Both viruses are transmitted through the respiratory tract, and it is possible to be protected with non-pharmacological interventions such as masks [32]. Measures were taken amid the COVID-19 pandemic, and a reduction in global travel has caused a decrease in the number of seasonal influenza cases. However, it is expected that influenza cases will rise as the level of international travel is slowly being restored. Distinguishing infection from SARS-CoV-2 vs. Influenza-A is clinically difficult in the early stages of infection. It is also important to keep in mind the possibility of co-infection, which can exacerbate the clinical prognosis. During the influenza season, it is difficult to confidently secure clinical diagnosis due to similar symptom presentations, such as fever, cough, and dyspnea. In this context, real-time polymerase chain reaction and nucleic acid tests can be performed on suspected patients to determine the viral etiology and institute appropriate treatment and, if applicable, isolation procedures. Our main motivation in this study was to create an accurate algorithm that can be used to classify infection due to SARS-CoV-2 versus Influenza-A at the highest level.

**Figure 5:**
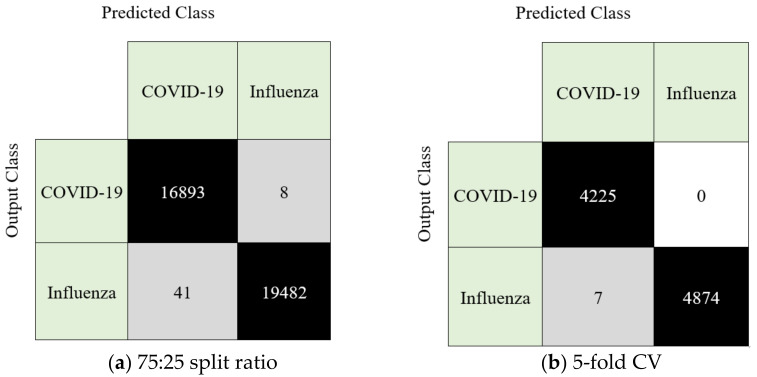
Confusion matrices of the HamletPat-based classification model using hold-out (split ratio 75:25) versus 5-fold cross-validations (CVs).

We presented a new handcrafted text-based feature generation model that could accurately discriminate between SARS-CoV-2 and Influenza-A. The novel *Hamlet* [33,34] Pattern is a local texture feature extractor with low time complexity, $O\left(n\right)$. IChi2 selected the most valuable 340 features among the 1280 features created with HamletPat, thereby effectively reducing the execution time. Using two validation techniques, these selected features were then classified using an SVM with a Gaussian kernel. The calculated confusion matrices according to the validation technique are shown in Figure 5.

The performance of our model for SARS-CoV-2 versus Influenza-A diagnosis is compared with another study in the literature that involved the binary classification of SARS-CoV-2 versus human immunodeficiency virus [9] in Table 3. The HamletPat-based model outperformed the other model on a larger dataset.

Table 3 shows that Afify and Zanaty [9] used a balanced protein sequence dataset with two classes, HIV and COVID-19. They achieved a 99.80% accuracy. We utilized an unbalanced dataset, and our classes are COVID-19 and Influenza-A (which have similar symptoms). Our proposal attained a 99.92% accuracy with a five-fold CV. Moreover, this model is the first text-based feature extractor generation methodology. The high classification capability of the features generated using HamletPat is clearly demonstrated. Moreover, we used a larger dataset than that of Afify and Zanaty’s [9]. This result demonstrates the robust feature generation ability of HamletPat.

**Figure 6:**
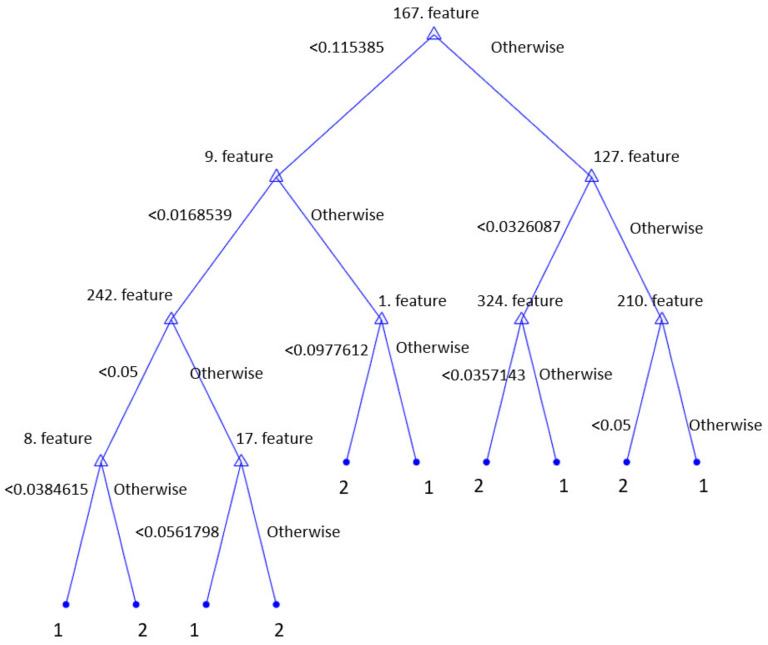
Rules of a basic decision-support system using our selected features. Herein, the symbol 1 denotes COVID-19, while 2 denotes influenza.

Moreover, using the presented HamletPat-based classification model, a decision-support system was created. By incorporating the 340 features selected, the decision support-system could be implemented. A graphical representation of the decision tree (pruning level = 10) for basic decision support is demonstrated in Figure 6.

By only using these nine rules (see Figure 6), 96.84% classification accuracy was attained on the used dataset. These nine rules demonstrated the explainability of the proposed system. By using HamletPat, features were created. Hidden rules were extracted by deploying the created features and a decision tree. These rules were created using features 167, 127, 210, 324, 9, 1, 242, 8, and 17 (see Figure 6). The proposed HamletPat is useful for creating a cognitive model to detect COVID-19. Figure 6 shows that an explainable artificial intelligence (XAI) model was proposed in this work. We were able to create a decision-support system by using these rules (see Figure 6), and this decision-support system can be embedded in a card to develop an embedded system.

The MATLAB codes of the presented pattern generation are given in Appendix A (Table A1 and Table A2).

The advantages and disadvantages of the novel HamletPat-based protein sequence classification model are listed below.

Advantages:

- Influenza and COVID-19 share similar symptoms, and clinical discrimination is difficult. Therefore, an automated protein-sequence-based model was developed to differentiate the disorders automatically.
- To our knowledge, HamletPat is the first text-based pattern utilized to create a new feature extraction function.
- The novel HamletPat-based classification model was trained on a two-class dataset and attained 99.87% and 99.92% accuracy rates by deploying a five-fold CV and hold-out (split ratio 75:25) CV, respectively.
- The model is simple, it has a low time complexity of $O\left(n\right),$ and is easy to implement.

Limitations:

- The model used overlapping blocks with a fixed length of 27. Therefore, the minimum length of the studied protein sequence should be 27 (we used a protein sequence with a length of 100 or greater in the study).
- We used the SVM classifier with default hyperparameters in the study. The hyperparameters can be further optimized using a metaheuristic optimization model.

## 5. Conclusions

To discriminate between SARS-CoV-2 and Influenza-A infections, we developed a new automated detection model for the binary classification of protein sequences. The handcrafted classification model used a textual-based pattern to extract 1280 features. IChi2 was used to select the 340 most discriminative features, and an SVM was used for classification using two validation strategies, hold-out (75:25 split ratio) and five-fold CV, which attained 99.92% and 99.87% accuracy rates, respectively. These results suggest that the proposed model has discriminative utility for the binary classification of SARS-CoV-2 vs. Influenza-A on the basis of protein sequences.

Developments in personalized or precision medicine have grown apace in recent years, and medical understanding is evolving with the use of new technologies. As a result, computer-assisted algorithms have become increasingly more relevant and valuable. We hope that our new textual-based feature generation methodology can lend assistance in this regard, and that new-generation explainable artificial intelligence models can be built to aid clinicians [35]. For now, the salutary results of our lightweight, handcrafted classification model suggest that it can be applied as a useful adjunctive screening tool to discriminate between these two important viral respiratory conditions.

## Figures and Tables

**Table 1:** Pattern array of the computed Hamlet Pattern.

id	1	2	3	4	5	6	7	8	9	10	11	12	13
Ind.	2	5	18	14	1	4	15	23	8	19	20	6	3
id	14	15	16	17	18	19	20	21	22	23	24	25	26
Ind.	9	25	13	21	12	7	22	11	16	17	24	10	26

Here “id” represents the identifying number of the enumerated letter (e.g., “1” and “26” represent “a” and “z”, respectively) and “Ind.” represents the corresponding computed index.

**Table 2:** Performance metrics for binary classification of viral protein sequences into SARS-CoV-2 versus Influenza-A using the HamletPat-based classification model.

Metric	Cross Validation	SARS-CoV-2	Influenza-A
**Sensitivity (%)**	5-fold CV	99.95	99.79
	75:25	100	99.86
**Specificity (%)**	5-fold CV	99.79	99.95
	75:25	99.86	100
**Precision (%)**	5-fold CV	99.76	99.96
	75:25	99.83	100
**F1-score (%)**	5-fold CV	99.86	99.87
	75:25	99.92	99.93
**Overall accuracy (%)**	5-fold CV	99.87	
	75:25	99.92	
**Overall geometric mean (%)**	5-fold CV	99.87	
	75:25	99.93	

**Table 3:** Comparative results (%).

Model	Dataset	Number of Observations	Method	Result
Afify and Zanaty [9]	NCBI	18,476 protein sequences: 9238 COVID-19 9238 HIV	Conjoint triad feature extraction and Random Forest classification with hold-out validation (80:20)	Accuracy: 99.80%
**Our model**	NCBI	36,424 protein sequences: 16,901 COVID-19 19,523 Influenza-A	HamletPat feature extraction, IChi2 feature selection, and SVM classification with hold-out validation (75:25) and 5-fold CV	Accuracy: hold-out: 99.92% 5-fold CV: 99.87%

## Data Availability

The data used in this study were downloaded from [22].

## Appendix A

We implemented the proposed model using the MATLAB (2021b) programming environment. The novelty of this paper is HamletPat. The MATLAB codes of the presented pattern generation of the text-based feature generation methodology and HamletPat are given in Table A1 and Table A2, respectively.

**Table A1:** Pattern generation code of the text-based feature generation methodology.

clc,clear all,close all text = ‘bernardowhostherefrancisconayanswermestandandunfoldyourselfbernardolonglivethekingfranciscobernardobernardohefranciscoyoucomemostcarefullyuponyourhourbernardotisnowstrucktwelvegettheetobedfranciscofranciscoforthisreliefmuchthankstisbittercoldandiamsickatheartbernardohaveyouhadquietguardfrancisconotamousestirringbernardowellgoodnightifyoudomeethoratioandmarcellustherivalsofmywatchbidthemmakehastefranciscoithinkihearthemstandhowhosthereenterhoratioandmarcellushoratiofriendstothisgroundmarcellusandliegementothedanefranciscogiveyougoodnightmarcellusofarewellhonestsoldierwhohathrelievedyoufranciscobernardohasmyplacegiveyougoodnightexitmarcellushollabernardobernardosaywhatishoratiotherehoratioapieceofhimbernardowelcomehoratiowelomegoodmarcellusmarcelluswhathasthisthingappeardagaintonightbernardoihaveseennothingmarcellushoratiosaystisbutourfantasyandwillnotletbelieftakeholdofhimtouchingthisdreadedsighttwiceseenofusthereforeihaveentreatedhimalongwithustowatchtheminutesofthisnightthatifagainthisapparitioncomehemayapproveoureyesandspeaktoithoratiotushtushtwillnotappearbernardositdownawhileandletusonceagainassailyourearsthataresofortifiedagainstourstorywhatwehavetwonightsseenhoratiowellsitwedownandletushearbernardospeakofthisbernardolastnightofallwhenyondsamestarthatswestwardfromthepolehadmadehiscoursetoillumethatpartofheavenwherenowitburnsmarcellusandmyselfthebellthenbeatingoneenterghostmarcelluspeacebreaktheeofflookwhereitcomesagainbernardointhesamefigurelikethekingthatsdeadmarcellusthouartascholarspeaktoithoratiobernardolooksitnotlikethekingmarkithoratiohoratiomostlikeitharrowsmewithfearandwonderbernardoitwouldbespoketomarcellusquestionithoratiohoratiowhatartthouthatusurpstthistimeofnighttogetherwiththatfairandwarlikeforminwhichthemajestyofburieddenmarkdidsometimesmarchbyheavenichargetheespeakmarcellusitisoffendedbernardoseeitstalksawayhoratiostayspeakspeakichargetheespeakexitghostmarcellustisgoneandwillntanswerbernardohownowhoratioyoutrembleandlookpaleisnotthissomethingmorethanfantasywhatthinkyouonthoratiobeforemygodimightnotthisbelievewithoutthesensibleandtrueavouchofmineowneyesmarcellusisitnotlikethekinghoratioasthouarttothyselfsuchwastheveryarmourhehadonwhenhetheambitiousnorwaycombatedsofrowndheoncewheninanangryparlehesmotethesleddedpolacksontheicetisstrangemarcellusthustwicebeforeandjumpatthisdeadhourwithmartialstalkhathhegonebyourwatchhoratioinwhatparticularthoughttoworkiknownotbutinthegrossandscopeofmyopinionthisbodessomestrangeeruptiontoourstatemarcellusgoodnowsitdownandtellmehethatknowswhythissamestrictandmostobservantwatchsonightlytoilsthesubjectofthelandandwhysuchdailycastofbrazencannonandforeignmartforimplementsofwarwhysuchimpressofshipwrightswhosesoretaskdoesnotdividethesundayfromtheweekwhatmightbetowardthatthissweatyhastedothmakethenightjointlabourerwiththedaywhoistthatcaninformmehoratiothatcaniatleastthewhispergoessoourlastkingwhoseimageevenbutnowappeardtouswasasyouknowbyfortinbrasofnorwaytheretoprickdonbyamostemulatepridedaredtothecombatinwhichourvalianthamletforsothissideofourknownworldesteemdhimdidslaythisfortinbraswhobyasealdcompactwellratifiedbylawandheraldrydidforfeitwithhislifeallthosehislandswhichhestoodseizedoftotheconqueroragainstthewhichamoietycompetentwasgagedbyourkingwhichhadreturndtotheinheritanceoffortinbrashadhebeenvanquisherasbythesamecovenantandcarriageofthearticledesigndhisfelltohamletnowsiryoungfortinbrasofunimprovedmettlehotandfullhathintheskirtsofnorwayhereandtheresharkdupalistoflawlessresolutesforfoodanddiettosomeenterprisethathathastomachintwhichisnootherasitdothwellappearuntoourstatebuttorecoverofusbystronghandandtermscompulsatorythoseforesaidlandssobyhisfatherlostandthisitakeitisthemainmotiveofourpreparationsthesourceofthisourwatchandthechiefheadofthisposthasteandromageinthelandbernardoithinkitbenootherbuteensowellmayitsortthatthisportentousfigurecomesarmedthroughourwatchsolikethekingthatwasandisthequestionofthesewarshoratioamoteitistotroublethemindseyeinthemosthighandpalmystateofromealittleerethemightiestjuliusfellthegravesstoodtenantlessandthesheeteddeaddidsqueakandgibberintheromanstreetsasstarswithtrainsoffireanddewsofblooddisastersinthesunandthemoiststaruponwhoseinfluenceneptunesempirestandswassickalmosttodoomsdaywitheclipseandeventhelikeprecurseoffierceeventsasharbingersprecedingstillthefatesandprologuetotheomencomingonhaveheavenandearthtogetherdemonstrateduntoourclimaturesandcountrymenbutsoftbeholdlowhereitcomesagainreenterghostillcrossitthoughitblastmestayillusionifthouhastanysoundoruseofvoicespeaktomeiftherebeanygoodthingtobedonethatmaytotheedoeasandgracetomespeaktomecockcrowsifthouartprivytothycountrysfatewhichhappilyforeknowingmayavoidospeakorifthouhastuphoardedinthylifeextortedtreasureinthewombofearthforwhichtheysayyouspiritsoftwalkindeathspeakofitstayandspeakstopitmarcellusmarcellusshallistrikeatitwithmypartisanhoratiodoifitwillnotstandbernardotisherehoratiotisheremarcellustisgoneexitghostwedoitwrongbeingsomajesticaltoofferittheshowofviolenceforitisastheairinvulnerableandourvainblowsmaliciousmockerybernardoitwasabouttospeakwhenthecockcrewhoratioandthenitstartedlikeaguiltythinguponafearfulsummonsihaveheardthecockthatisthetrumpettothemorndothwithhisloftyandshrillsoundingthroatawakethegodofdayandathiswarningwhetherinseaorfireinearthorairtheextravagantanderringspirithiestohisconfineandofthetruthhereinthispresentobjectmadeprobationmarcellusitfadedonthecrowingofthecocksomesaythatevergainstthatseasoncomeswhereinoursavioursbirthiscelebratedthebirdofdawningsingethallnightlongandthentheysaynospiritdaresstirabroadthenightsarewholesomethennoplanetsstrikenofairytakesnorwitchhathpowertocharmsohallowdandsograciousisthetimehoratiosohaveiheardanddoinpartbelieveitbutlookthemorninrussetmantlecladwalksoerthedewofyonhigheastwardhillbreakweourwatchupandbymyadviceletusimpartwhatwehaveseentonightuntoyounghamletforuponmylifethisspiritdumbtouswillspeaktohimdoyouconsentweshallacquainthimwithitasneedfulinourlovesfittingourdutymarcellusletsdotiprayandithismorningknowwhereweshallfindhimmostconveniently’; number = double(text)-96; histo = zeros(1,26); for j = 1:length(number) histo(number (j)) = histo(number (j)) + 1; end plot(histo) % Pattern Generation counter = zeros(1,26); summ = sum(counter); i = 1; j = 1; while(summ < 26) sy = number(i); if (counter(sy) == 0) counter(sy) = 1; pattern(j) = sy; j = j + 1; end summ = sum(counter); i = i + 1; end

**Table A2:** The proposed HamletPat.

function histo = hamlet_pat(sinyal) h1 = zeros(1512); h2 = zeros(1256); h3 = h1; for i = 1:length(sinyal)-26 blok = sinyal(i:i + 26); m = blok(14); deger(1:13) = blok(1:13); deger(14:26) = blok(15:27); for j = 1:26 bit(j) = deger(j) >= m; end b1(1:9) = bit(1:9); b2(1:8) = bit(10:17); b3(1:9) = bit(18:26); m1(i) = 0; m2(i) = 0; m3(i) = 0; for j = 1:9 m1(i) = m1(i) + b1(j)*2^(j-1); m3(i) = m3(i) + b3(j)*2^(j-1); end for j = 1:8 m2(i) = m2(i) + b2(j)*2^(j-1); end h1(m1(i) + 1) = h1(m1(i) + 1) + 1; h2(m2(i) + 1) = h2(m2(i) + 1) + 1; h3(m3(i) + 1) = h3(m3(i) + 1) + 1; end histo = [h1 h2 h3];
